# Reconstruction and analysis of a genome-scale metabolic model of the oleaginous fungus *Mortierella alpina*

**DOI:** 10.1186/s12918-014-0137-8

**Published:** 2015-01-13

**Authors:** Chao Ye, Nan Xu, Haiqin Chen, Yong Q Chen, Wei Chen, Liming Liu

**Affiliations:** State Key Laboratory of Food Science and Technology, Jiangnan University, 1800 Lihu Road, Wuxi, Jiangsu 214122 China; The Key Laboratory of Industrial Biotechnology, Ministry of Education, Jiangnan University, 1800 Lihu Road, Wuxi, Jiangsu 214122 China; Synergistic Innovation Center for Food Safety and Nutrition, School of Food Science and technology, Jiangnan University, 1800 Lihu Road, Wuxi, Jiangsu 214122 China

**Keywords:** *Mortierella alpina*, Arachidonic acid, Genome-scale metabolic model, Polyunsaturated fatty acids, Malic enzyme

## Abstract

**Background:**

*Mortierella alpina* is an oleaginous fungus used in the industrial scale production of arachidonic acid (ARA). In order to investigate the metabolic characteristics at a systems level and to explore potential strategies for enhanced lipid production, a genome-scale metabolic model of *M. alpina* was reconstructed.

**Results:**

This model included 1106 genes, 1854 reactions and 1732 metabolites. On minimal growth medium, 86 genes were identified as essential, whereas 49 essential genes were identified on yeast extract medium. A series of sequential desaturase and elongase catalysed steps are involved in the synthesis of polyunsaturated fatty acids (PUFAs) from acetyl-CoA precursors, with concomitant NADPH consumption, and these steps were investigated in this study. Oxygen is known to affect the degree of unsaturation of PUFAs, and robustness analysis determined that an oxygen uptake rate of 2.0 mmol gDW^−1^ h^−1^ was optimal for ARA accumulation. The flux of 53 reactions involving NADPH was significantly altered at different ARA levels. Of these, malic enzyme (ME) was confirmed as a key component in ARA production and NADPH generation. When using minimization of metabolic adjustment, a knock-out of ME led to a 38.28% decrease in ARA production.

**Conclusions:**

The simulation results confirmed the model as a useful tool for future research on the metabolism of PUFAs.

**Electronic supplementary material:**

The online version of this article (doi:10.1186/s12918-014-0137-8) contains supplementary material, which is available to authorized users.

## Background

*Mortierella alpina* is an oleaginous zygomycete, that can accumulate lipids to 50% of its dry weight in the form of triacylglycerols [1]. The important ω-6 polyunsaturated fatty acid (PUFA) arachidonic acid (ARA) can account for over 50% of the lipid content [2]. *M. alpina* is nonpathogenic and nonallergenic, including the spores produced during the industrial production of ARA [3] which is widely used in food ingredients [4]. ARA has been produced at levels up to 19.8 g/L in 5 L cultures grown over 7 days [5]. Various methods have been attempted in order to improve ARA production including screening potentially higher yielding mutant strains following treatment with *N*-methyl-*N*’-nitro-*N*-nitrosoguanidine (MNNG) [6]. This work led to the generation of *M. alpina* strain Y11 which possessed lowered ω-3 desaturation activity, and ARA production was 2.1 fold (2.21 g/L) higher than strain 1S-4. Optimizing the culture medium and fixing the ratio of defatted soybean meal to potassium nitrate at 2:1 gave a fourfold increase (6.0 g/L) in ARA production [7]. In another study the fermentation process was optimized, and a two-stage temperature-shift strategy increased ARA production by 26.1% (9.2 g/L) [8]. Additionally, overexpressing GLELO gene using a genetic manipulation approach increased ARA production by 101.2% (5.05 g/L) [9].

Genetic engineering of *M. alpina* for enhanced ARA production remains an attractive proposition and for further research. Early work identified desaturases as key enzymes in PUFA synthesis. Specifically, Δ5 desaturase [10], which catalyzes dehydrogenation of dihomo-γ-linolenic acid (DGLA) to form ARA, was isolated and functionally characterized. The rate-limiting step for ARA biosynthesis is catalyzed by elongase which converts γ-linolenic acid (GLA) to DGLA [11,12]. NADH-cytochrome b5 reductase (Cb5R), an electron carrier and a major component of the cytochrome b5-dependent electron transport system, is also crucial. Cb5R catalyzes several different reduction reactions, including the desaturation and elongation of acyl chains built from acetyl-CoA during PUFA synthesis [13]. Despite various studies that have identified the importance of these enzymes in PUFA synthesis and metabolism [2,14], their exact roles are not completely understood, and neither are the pathways through which glucose relates to PUFA biosynthesis and metabolism.

A genome-scale metabolic model (GSMM) is an indispensable tool for the study of metabolism that adopts a systems biology approach to integrate data from genomics, transcriptomics, proteomics and metabolomics. It has been widely used in the analysis of the network properties of metabolism [15], prediction and analysis of organism growth phenotypes [16], model-based interpretation of experimental data [17], and metabolic engineering [18]. Oleaginous organisms such as *M. alpina* can accumulate large quantities of lipids, but maximizing lipid production is complicated by the complexity of the regulatory mechanisms associated with lipid metabolism. It is generally difficult to identify key metabolic modules contributing to lipid physiology. Using reconstruction GSMM, we can systematically analyze the function of each gene and metabolic reaction and model the effects using flux balance analysis (FBA). Specific pathways can be understood based on the model of the whole metabolic network, and strain design strategies can also be used to guide metabolic engineering experiments. Two GSMM studies on *Yarrowia lipolytica* (*i*NL895 [19] and *i*YL619_PCP [20]) have been published along with recent modeled networks of *Mucor circinelloides* and *M. alpina* [21]. GSMM studies therefore provide a new approach to investigating the complex lipid metabolism in *M. alpina*. Vongsangnak *et al*. (2013) [21] previously published a *M. alpina* network model, however, this was a refined network that could only be used to investigate genome annotation and metabolic routes, and not flux distribution or phenotypic behaviors [21]. To systematically study flux distribution and the mechanism of lipid accumulation, we reconstructed a new *M. alpina* GSMM and used the COBRA Toolbox [22] for subsequent research.

In this study, a genome-scale metabolic model (*i*CY1106) of *M. alpina* ATCC 32222 was reconstructed based on sequencing results [2]. Using this model, we first identified essential genes and reactions in fermentation medium containing sodium nitrate as a nitrogen source [23]. The *de novo* synthesis pathways of PUFAs such as ARA and eicosapentaenoic acid (EPA) were subsequently resolved. The roles of acetyl-CoA and NADPH in the regulation of PUFA biosynthesis were probed, and important genes and reactions were systematically analyzed using FBA, flux variability analysis (FVA), and minimization of metabolic adjustment (MOMA).

## Results and discussions

### Characterization of the *M. alpina* GSMM *i*CY1106

The genome-scale metabolic model *i*CY1106 reconstructed in this study included 1106 genes representing 9.51% of the 11631 protein-coding genes in the genome of *M. alpina* ATCC 32222. The model consisted of 1854 reactions and 1732 metabolites (Table 1). Compared with the previous model constructed by Vongsangnak [21], *i*CY1106 is more detailed and contains a higher degree of functional information that can be used in *in silico* simulations. During the construction of *i*CY1106, some missing gene were added from the Vongsangnak’s model such as MA-090-247, which encodes an asparaginase (EC: 3.5.1.1), and catalyzes the transformation of L-asparagine into L-aspartate. When compared with the existing model, many improvements in *i*CY1106 are apparent. (1) A total of 139 transport proteins were annotated based on TCDB and TransportDB. (2) A total of 216 exchange reactions were added into model *i*CY1106. (3) Biomass composition of *M. alpina* was calculated based on literature mining. (4) Additional chemical reactions were also included. (5) The reversibility of some reactions was redefined according to MetaCyc and BioPath databases. For example, in the published model, the reaction catalyzed by aldehyde dehydrogenase (EC: 1.2.1.3) was reversible, whereas this reaction is irreversible according to BioPath. The flux distribution of *i*CY1106 was normal following this change.

**Table 1:** **General features of model** ***i*** **CY1106**

**Features**	***i*** **CY1106**	**Existed model** ^**a**^
Genome feature		
Genome size (Mb)	38.38	38.38
Total open reading frames (ORFs)	11631	11631
Metabolic model		
Total reactions	1854	1183
Biochemical reactions	1391	1183
Transport reactions	247	none
Exchange reactions	216	none
Metabolites	1732	1660
ORFs associated in model	1106	1042
ORF coverage^b^ (%)	9.51	8.95

^a^The existed model was constructed by Vongsangnak et al. [21]; ^b^The number of ORFs incorporated in model *i*CY1106 divided by the total number of ORFs.

In model *i*CY1106, four compartments (extracellular, cytoplasmic, peroxisome and mitochondrial compartments) were linked by 141 trans-plasma membrane transport reactions, 98 cytoplasmic-mitochondrial transport reactions and eight cytoplasmic-peroxisome transport reactions (Additional file 1). The extracellular compartment contained 267 reactions, including 216 exchange reactions and 51 metabolic reactions. These metabolic reactions catalyzed by extracellular enzymes may enable *M. alpina* to adapt to different environmental conditions. For example, *M. alpina* grows on different carbohydrates such as glycerol [24], sucrose [24], d-mannose [25], and raffinose [25] using various extracellular galactosidases. The peroxisome compartment contained 34 reactions mostly related to glyoxylate metabolism.

**Figure 1:**
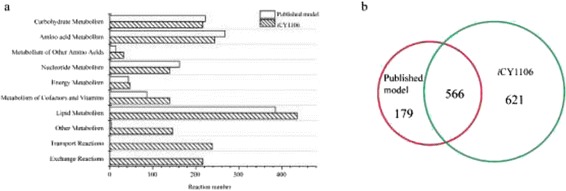
**The comparison between Vongsangnak’s model and** ***i*** **CY1106. a**: reaction distribution in subsystems between these two models; **b**: shared reactions and unique reactions between models.

Metabolic reactions in the model *i*CY1106 were divided into ten metabolic subsystems (Figure 1). The largest subsystem was lipid metabolism (fatty acid biosynthesis, fatty acid elongation, steroid, glycerolipid, glycerophospholipid and sphingolipid metabolism) that accounted for 23.53% of the total reactions. Reactions involving the synthesis of PUFAs such as DGLA, ARA, and EPA are listed in model *i*CY1106. For most subsystems, most categories were more abundant in *i*CY1106 than in the Vongsangnak’s model, with the exception of Amino Acid Metabolism and Nucleotide Metabolism (Figure 1a). Since there were six compartments (cytosol, mitochondria, peroxisome, extracellular, plasma membrane and unclassified compartment) in Vongsangnak’s model, some reactions occur in multiple compartments at the same time. When ignoring compartments, not including transport reactions and exchange reactions, these two models shared 566 reactions (Additional file 2). Vongsangnak’s model and *i*CY1106 included 179 and 621 unique reactions, respectively (Figure 1b). Compared with model *i*YL619_PCP of the oleaginous yeast *Y. lipolytica* [20], lipid metabolism accounted for a higher proportion (16.7%, 191 of total 1142 reactions) of reactions in *i*CY1106. Amino acid and carbohydrate metabolism subsystems were 13.17% and 11.66%, respectively, in *i*CY1106.

### Identification of essential genes and reactions in *i*CY1106

**Figure 2:**
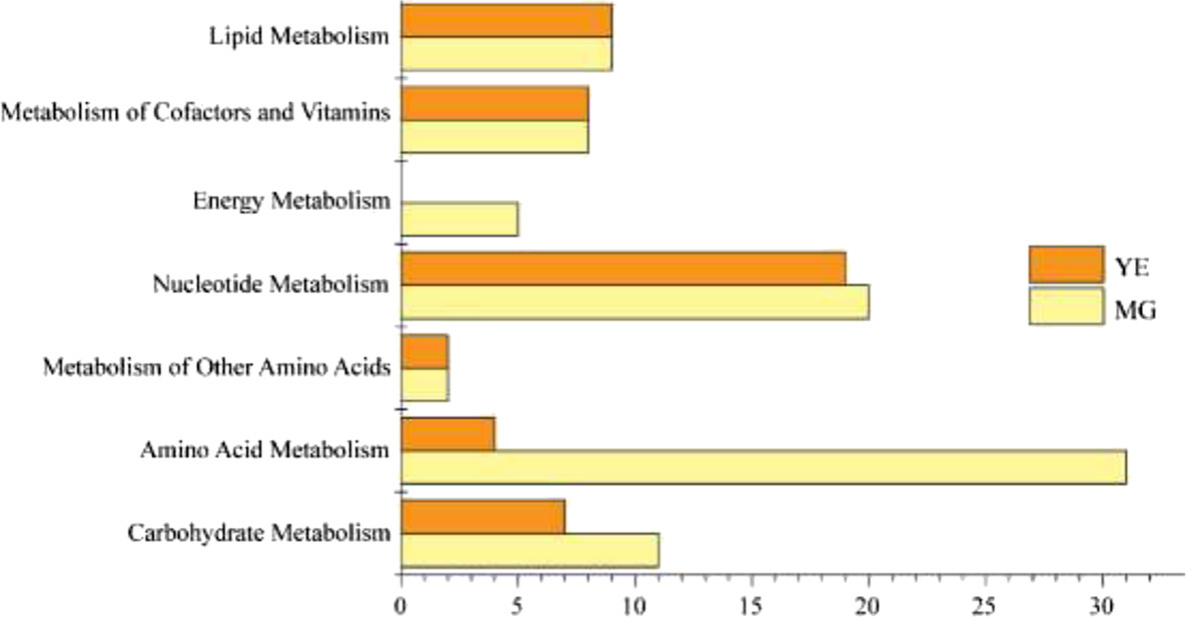
**Essential genes identified by MG and YE medium in model** ***i*** **CY1106.**

When cultured on minimal growth medium (MG) containing glucose, KH_2_PO_4_, MgSO_4_ and NaNO_3_ [23], 86 *M. alpina* genes (7.78% of the total) were identified as essential for growth using the FBA method (Additional file 3). In contrast, on yeast extract (YE) medium (based on MG but including all 20 regular amino acids) only 49 genes were identified as essential. On MG medium, over half of the essential genes were involved in amino acid (36.05%) and nucleotide metabolism (23.26%; Figure 2). However on YE medium, most of the essential genes were associated with nucleotide metabolism (38.78%).

The identified genes were compared with the database of essential genes (DEG), and 40 genes were found to share high homology with genes identified by DEG (identity ≥ 40%, E-value ≤ 1.0E-30; Table 2). One gene, UTP-glucose-1-phosphate uridylyltransferase (MA-090-485), is involved in the synthesis of the cell wall component 1,3-beta-D-glucan (Table 2). MA-090-485 is highly homologous with the *Saccharomyces cerevisiae* enzyme (sequence identity = 62.58%, e-value = 0) identified in the DEG search. Similarly, nucleoside diphosphate kinase (MA-120-261), which is involved in the synthesis of CTP and GTP, also shared high homology with one of the essential genes identified by DEG (identity = 66.89%, e-value = 2.0E-68). Besides, in MG, there were 46 genes that did not match with DEG (Additional file 3). Of these, when nitrate was used as nitrogen source, nitrate reductase (MA-291-2) and nitrite reductase (MA-291-3), which together convert nitrate into NH_3_ for cell growth, were essential under this condition. Another two essential genes, delta 5 desaturase (MA-326-160) and delta 12 desaturase (MA-334-414) are vital for the synthesis of ARA in *M. alpina*. In addition, most of these essential genes were distributed in the Amino Acid Metabolism category (28.3%). This was understandable since essential gene identified by DEG were in a rich medium in which genes involving amino acid synthesis may not be essential.

**Table 2:** **Essential genes identified on different mediums which are high homology with DEG**

**Gene**	**Enzyme**	**Essentiality**		**Subsystem**
		**MG**	**YE**	
MA-133-4	Ribose-5-phosphate isomerase	E	E	Carbohydrate metabolism
MA-090-485	UTP-glucose-1-phosphate uridylyltransferase	E	E	
MA-101-200	Mannose-6-phosphate isomerase	E	E	
MA-120-141	Phosphoacetylglucosamine mutase	E	E	
MA-184-380	Acetyl-CoA carboxylase	E	E	
MA-072-196	Argininosuccinate lyase	E	NE	Amino acid metabolism
MA-213-50	Adenylosuccinate synthase	E	E	
MA-213-539	Adenylosuccinate lyase	E	E	
MA-326-6	Aspartate carbamoyltransferase	E	E	
MA-334-434	Phosphoribosylanthranilate isomerase	E	NE	
MA-120-148	2-Acetolactate methylmutase	E	NE	
MA-090-434	Dihydroxy-acid dehydratase	E	NE	
MA-139-331	Homoaconitate hydratase	E	NE	
MA-323-77	Saccharopine dehydrogenase	E	NE	
MA-326-106	Ornithine carbamoyltransferase	E	NE	
MA-101-393	Imidazole-4-carboxamide isomerase	E	NE	
MA-184-558	Imidazoleglycerol-phosphate dehydratase	E	NE	
MA-090-452	Glutamine amidotransferase:cyclase	E	NE	
MA-320-159	3-dehydroquinate synthase	E	NE	
MA-139-157	Chorismate synthase	E	NE	
MA-073-62	3-deoxy-7-phosphoheptulonate synthase	E	NE	
MA-213-547	Tryptophan synthase	E	NE	
MA-297-40	Chorismate mutase	E	NE	
MA-120-157	Thioredoxin reductase	E	E	
MA-173-30	Purine-nucleoside phosphorylase	E	E	Nucleotide metabolism
MA-213-65	IMP dehydrogenase	E	E	
MA-120-261	Nucleoside diphosphate kinase	E	E	
MA-139-347	Guanylate kinase	E	E	
MA-120-138	Ribonucleoside-diphosphate reductase	E	E	
MA-055-211	Ribonucleoside-diphosphate reductase	E	E	
MA-323-58	Ribonucleoside-diphosphate reductase	E	E	
MA-334-356	Thymidylate synthase	E	E	
MA-153-455	3(2),5-bisphosphate nucleotidase	E	NE	
MA-111-23	phosphoadenylyl-sulfate reductase	E	NE	Energy metabolism
MA-182-360	6,7-dimethyl-8-ribityllumazine synthase	E	E	Cofactors and vitamins
MA-184-368	Riboflavin synthase	E	E	
MA-210-311	Aspartate dehydrogenase	E	E	
MA-055-340	Fatty-acyl-CoA synthase	E	E	Lipid metabolism
MA-162-131	Palmitoyl-protein thioesterase	E	E	
MA-334-239	Very-long-chain enoyl-CoA reductase	E	E	

*E*: essential gene; *NE*: non-essential gene. *MG*: minimal medium; *YE*: yeast extract medium.

### Simulation and verification of model *i*CY1106

FBA with constraints was used to investigate the metabolic properties of model *i*CY1106. YE medium was used to simulate the growth rate and ARA production, the maximum uptake rate of all 20 amino acids were set at 0.01 mmol gDW^−1^ h^-1^ [26], and the glucose consumption rate was set at 0.8 mmol gDW^−1^ h^-1^ [8], and 2.0 mmol gDW^−1^ h^-1^ [1] respectively, and the *in silico* growth rate were closed to experimental values (differences were only 0.8% and 9.48%; Table 3). To investigate ARA production *in silico*, the growth rate was fixed at 0.03 h^-1^ [8], and the ARA exchange reaction was set as the objective function. Under these conditions, the ARA rate was 0.128 mmol gDW^−1^ h^−1^ by FBA. This *in silico* value was lower than the experimentally determined value of 0.149 mmol gDW^−1^ h^−1^ (14.09%) [8], and this may due to the use of two different *M. alpina* strains (ATCC 32222 and ME-1) for modeling and experimental components, with ME-1 being the higher ARA producing strain [27]. Furthermore, the difficulties associated with accurately modeling the yeast extract may have resulted in a nutritional deficiency in the model.

**Table 3:** **Comparison of** ***in silico*** **and** ***in vivo*** **growth rates of** ***M*** **.** ***alpina***

**Media condition (mmol gDW** ^**−1**^ **h** ^**−1**^ **)**	**Growth rate (h** ^**−1**^ **)**		**Ref.**
	***in vivo***	***in silico***	
Glc (v = 0.8)	0.0696	0.0690	[8]
Glc (v = 2.0)	0.1708	0.1546	[1]

*in vivo*: experimental results; *in silico*: simulation results.

The capability of utilizing 14 different carbon sources for cell growth was qualitatively predicted by the model *i*CY1106 using FBA (Additional file 4). Each substance was the sole carbon source and the rate of uptake was 1.0 mmol gDW^−1^ h^−1^. Initially, there were four types of carbon source (ethanol, xylose, maltose, and rhamnose), and the model could not achieve growth with any individual carbon source. For ethanol, the reaction catalyzed by alcohol dehydrogenase (EC: 1.1.1.1) was irreversible, but should be reversible according to the MetaCyc database. For rhamnose, the lack of L-rhamnulose-1-phosphate lactaldehyde-lyase (EC: 4.1.2.19) meant that rhamnose could not generate glycerone phosphate, which could otherwise be used by the glycolysis pathway. After elimination of initial discrepancies using the continuous gap filling process, a 100% match was acquired. This indicated that model *i*CY1106 could predict catabolic pathways of various carbon sources including common sugars and alcohols. A similar FBA simulation of nitrogen sources (ammonium, nitrate, urea, and amino acids) also generated results that were consistent with experimental fermentation data. Both glutamate and glycine supported *M. alpina* growth, confirming that the model performed well for predicting the utilization of different carbon and nitrogen sources.

### PUFA biosynthetic pathways based on GSMM

**Figure 3:**
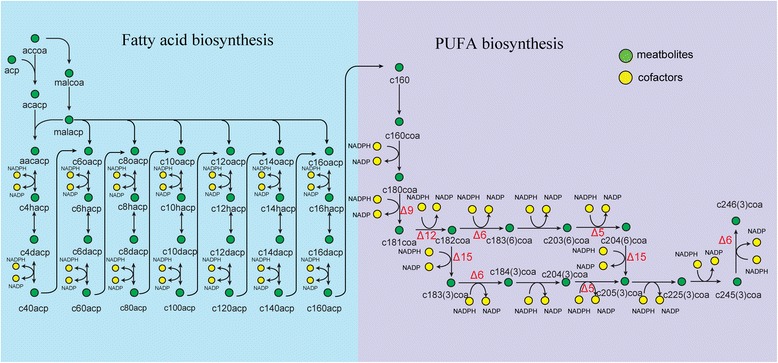
**Synthesis pathway of PUFAs in** ***M*** **.** ***alpina*** **.** (accoa: acetyl-CoA, acp: acyl-carrier protein, acacp: Acetyl-[acyl-carrier protein], malcoa: malonyl-CoA, malacp: malonyl-[acp], aacacp: acetoacetyl [acp], c4hacp: (3R)-3-Hydroxybutanoyl-[acp], c4dacp: but-2-enoyl-[acp], c40acp: butanoyl-[acp], c6oacp: 3-oxohexanoyl-[acp], c6hacp: 3-hydroxyhexanoyl-[acp], c6dacp: hex-2-enoyl-[acp], c60acp: hexanoyl-[acp], c8oacp: 3-oxooctanoyl-[acp], c8hacp: 3-hydroxyoctanoyl-[acp], c8dacp: oct-2-enoyl-[acp], c80acp: octanoyl-[acp], c10oacp: 3-oxodecanoyl-[acp], c10hacp: 3-hydroxydecanoyl-[acp], c10dacp: dec-2-enoyl-[acp], c100acp: decanoyl-[acp], c12oacp: 3-oxododecanoyl-[acp], c12hacp: 3-hydroxy-dodecanoyl-[acp], c12dacp: dodec-2-enoyl-[acp], c120acp: dodecanoyl-[acp], c14oacp: 3-oxomyristoyl-[acp], c14dacp: tetradec-2-enoyl-[acp], c14hacp: 3-hydroxymyristoyl-[acp], c140acp: myristoyl-[acp], c16oacp: 3-oxohexadecanoyl-[acp], c16hacp: (3R)-3-Hydroxypalmitoyl-[acp], c16dacp: hexadec-2-enoyl-[acp], c160acp: hexadecanoyl-[acp], c160: palmitic acid, c160coa: hexadecanoyl-CoA, c180coa: octadecanoyl-CoA, c181coa: octadecenoyl-CoA, c182coa: linoleoyl-CoA, c183(6)coa: gamma-Linolenoyl-CoA, c203(6)coa: 8,11,14-Eicosatrienoyl-CoA, c204(6)coa: Arachidonyl-CoA, c204(6): ARA, c183(3)coa: alpha-Linolenoyl-CoA, c184(3)coa: Stearidonoyl-CoA, c204(3)coa: (11Z,14Z,17Z)-Icosatrienoyl-CoA, c205(3)coa: eicosapentaenoyl-CoA, c225(3)coa: (7Z,10Z,13Z,16Z,19Z)-Docosapentaenoyl-CoA, c245(3)coa: (9Z,12Z,15Z,18Z,21Z)-Tetracosaheptaenoyl-CoA, c246(3)coa: (6Z,9Z,12Z,15Z,18Z,21Z)-Tetracosahexaenoyl-CoA, c226(3)coa: (4Z,7Z,10Z,13Z,16Z,19Z)-Docosahexaenoyl-CoA).

To further investigate the pathways associated with PUFA biosynthesis and metabolism, genome annotation, literature mining and KEGG database analysis were performed. ARA is a ω-6 PUFA synthesized by *M. alpina* (Figure 3). ARA (c204(6)) is synthesized from acetyl-CoA in 38 enzymatic steps, including the *de novo* synthesis of palmitic acid (c160) and the synthesis of very long chain fatty acids. During PUFA biosynthesis, all fatty acids should be in the form of acyl-CoA [2]. Desaturase and elongase were necessary for the conversion of octadecanoyl-CoA (c180coa) to ARA. Δ9 desaturase [28] (MA-055-51, MA-101-533, MA-184-235), Δ12 desaturase [29] (MA-334-414) and Δ6 desaturase [30,31] (MA-268-54, MA-101-36) catalyzed the conversion of c180coa into γ-linolenoyl-CoA (c183(6)coa) which was subsequently lengthened to 8,11,14-eicosatrienoyl-CoA (c203(6)coa) by an elongase such as ELO/GLELO [30] (MA-189-257), MAELO [32] (MA-184-206) or MALCE1 [33] (MA-073-327, MA-320-221). Δ5 desaturase [10] (MA-326-160) catalyzed c203(6)coa to arachidonyl-CoA (c204(6)coa), which was finally hydrolyzed to c204(6) by choloyl-CoA hydrolase (MA-049-4, MA-139-47).

*M. alpina* is capable of producing ARA and EPA, and EPA can be produced via the ω-3 or the ω-6 PUFA biosynthetic pathways through ARA desaturation [34]. During EPA biosynthesis, Δ15 (ω3) desaturase (MA-072-10) is the key enzyme and was the first fungal desaturase known that uses both 18-carbon and 20-carbon ω-6 PUFAs as substrates [35]. In the ω-3 pathway, (11Z,14Z,17Z)-icosatrienoyl-CoA (c204(3)coa) was used as substrate, while the ω-6 pathway utilized c204(6)coa.

Both the maximum and minimum flux of oxygen absorption of DGLA, ARA and EPA production *in silico* were found to be 1.552, 1.674 and 1.891 mmol gDW^−1^ h^−1^ respectively, using FVA. This indicated that the degree of PUFA unsaturation was dependent on oxygen content. Robustness analysis was used to elucidate the effects of oxygen uptake rate on PUFA biosynthesis. The oxygen uptake rate was perturbed by constraints-based flux analysis between 0 and 20 mmol gDW^−1^ h^−1^. The minimum growth rate was set at 0.03 h^−1^, with ARA exchange reaction as the objective function. When oxygen absorption was lower than 2.0 mmol gDW^−1^ h^−1^, ARA production increased with oxygen absorption rate (Additional file 5a). However, beyond this range, ARA production gradually declined with increasing oxygen uptake rate. As reported by Higashiyama et al., when the DO concentration range was 10-15 ppm, the ARA yield was enhanced 1.6-fold over a DO of 7 ppm, and a DO between 20 and 50 ppm drastically decreased ARA production [36]. This indicated that excessive oxygen exposure during fermentation could impair ARA overproduction. During ARA biosynthesis, high oxygen concentrations may affect metabolism and cell growth in the filamentous mycelia, and beta-oxidation of fatty acids may be needed to obtain the extra energy required for adaptation to the high oxygen conditions.

### Regulation of PUFA biosynthesis and metabolism based on GSMM

**Figure 4:**
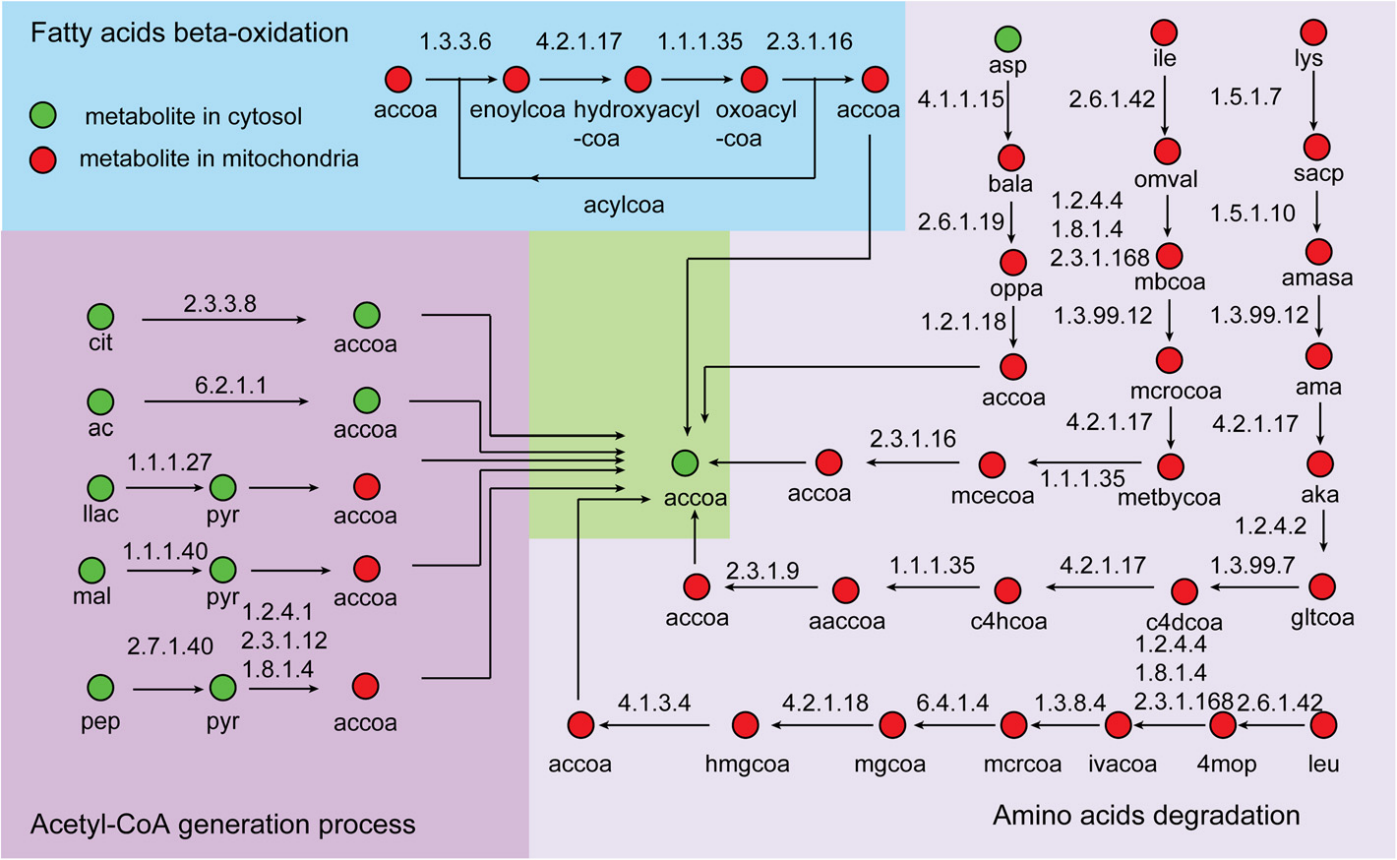
**The sources of acetyl-CoA in** ***M*** **.** ***alpina*** **.** (cit: citrate, ac: acetate, llac: l-lactate, mal: malate, pep: phosphoenolpyruvate, pyr: pyruvate, accoa: acrtyl-CoA, asp: l-aspartate, bala: beta-alanine, oppa: 3-oxopropanoate, ile: l-isoleucine, omval: (s)-3-nethyl-2-oxopentanoate, mbcoa: 2-methylbutanoyl-CoA, mcrocoa: 2-methylcrotonoyl-CoA, metbycoa: (2S,3S)-3-Hydroxy-2-methylbutanoyl-CoA, mcecoa: 2-Methylacetoacetyl-CoA, lys: l-lysine, sacp: l-saccharopine, amasa: l-2-aminoadipate 6-semialdehyde, ama: l-2-aminoadipate, aka: 2-oxoadipate, gltcoa: glutaryl-CoA, c4dcoa: crotonoyl-coa, c4hcoa: (S)-3-Hydroxybutanoyl-CoA, aaccoa: acetoacetyl-CoA, leu: l-leucine, 4mop: 4-methyl-2-oxopentanoate, ivacoa: 3-isovaleryl-coa, mcrcoa: 3-nethylcrotonyl-CoA, mgcoa: trans-3-methylglutaconyl-CoA, hmgcoa: (s)-3-hydroxy-3-methylglutaryl-coa).

Acetyl-CoA is the essential two-carbon donor molecule for fatty acid synthesis. In model *i*CY1106, there were ten pathways that could produce acetyl-CoA, including amino acid degradation (from aspartate, isoleucine, leucine, and lysine), fatty acid beta-oxidation, and other acetyl-CoA generation processes (phosphoenolpyruvate [PEP], malate, L-lactate, acetate, and citrate) (Figure 4). However, only the pathway from PEP to pyruvate could generate sufficient flux for synthesis of PUFAs on the fermentation medium. During the growth stage, the flux of acetyl-CoA generated by pyruvate was 1.22 mmol gDW^−1^ h^−1^, while this value was 1.56 mmol gDW^−1^ h^−1^ during the product synthesis stage. This corresponds to a 27.87% increase, which would ensure acetyl-CoA was available for ARA production due to the enhanced activity of the pyruvate dehydrogenase complex (EC: 1.2.4.1, 2.3.1.12, 1.8.1.4) [37]. Although aspartate, isoleucine, leucine, and lysine could all be used to generate acetyl-CoA for ARA production. Asparate has four carbon, while isoleucine, leucine, and lysine all contains six carbon. When the maximum uptake rate of asparate and the other amino acids were set at 0.15 mmol gDW^−1^ h^−1^ and 0.1 mmol gDW^−1^ h^−1^, respectively. FBA showed only that leucine and lysine could increase ARA production by 14.06% and 13.28%.

In model *i*CY1106, acetyl-CoA can be consumed by amino acid synthesis, amino sugar metabolism and fatty acid synthesis as well as ARA production (Additional file 5b). During the synthesis of six amino acids (threonine, methionine, lysine, cysteine, ornithine, and leucine), acetyl-CoA was required. FBA results showed that during the growth stage, 96.5% of the acetyl-CoA flux was used to synthesize fatty acids and 2.58% was used for amino acid synthesis. In contrast, during the product synthesis stage, the flux of acetyl-CoA used for fatty acid synthesis accounted for 99.52%, which indicates that amino acid synthesis was inhibited during ARA production. Limiting the nitrogen source can be an effective strategy to control amino acids biosynthesis [38], in which the lipid yield may be increased despite decreases in mycelia concentration.

During the growth stages, the flux of acetyl-CoA used to synthesize malonyl-CoA, catalyzed by acetyl-CoA carboxylase (ACC, EC 6.4.1.2), was 0.79 mmol gDW^−1^ h^−1^. In contrast during the ARA production stage, the corresponding flux was 1.43 mmol gDW^−1^ h^−1^ (81.0%). ACC catalyzes the first committal step of the fatty acid biosynthetic pathway, and should be overexpressed to maximize ARA production [39]. Glutamate has been shown to activate ACC, and adding glutamate to the culture medium led to an increase in total lipid (21.81%) and ARA yield (66.07%) [40]. As the *M. alpina* growth rate in batch culture ranged from 0.06 h^−1^ to 0.19 h^-1^ [1,7,8,41,42], the glutamate uptake rates used for the *in silico* analysis should be set between 0 and 1.8 mmol gDW^−1^ h^−1^. When the glutamate uptake rate was increased, ARA production increased from 0.128 mmol gDW^−1^ h^−1^ to 0.355 mmol gDW^−1^ h^−1^ (Additional file 5c).

**Figure 5:**
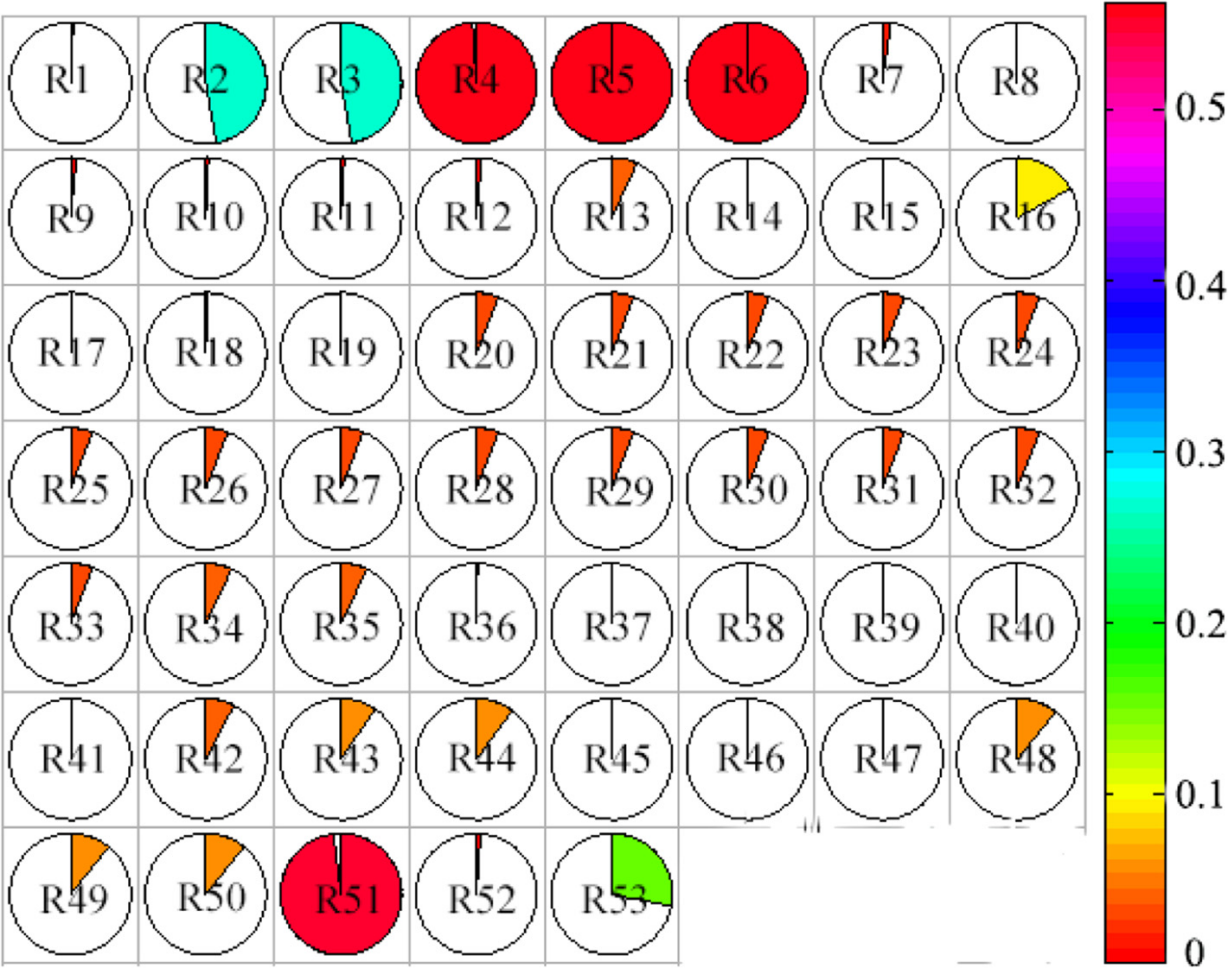
**Changes of reactions involving NADPH at different ARA levels.** (Different colors represent different changes on NADPH flux. Flux obviously changed reactions are listed. R2: D-glucose 6-phosphate + NADP ⬄ 6-phospho-D-glucono-1,5-lactone + NADPH + H, R3: 6-phospho-D-gluconate + NADP - > D-ribulose 5-phosphate + CO2 + NADPH + H, R4: (S)-malate + NADP - > pyruvate + CO2 + NADPH, R5: L-glutamate 5-semialdehyde + NAD P+ H2O - > L-glutamate + NADPH + H, R6: 1-pyrroline-5-carboxylate + NADPH + H ⬄ L-proline + NADP, R16: Nitrite + 3 NADPH + 5 H - > Ammonia + 3 NADP + 2 H2O, R51: N-acetyl-L-glutamyl 5-phosphate + NADPH + H ⬄ N-acetyl-L-glutamate 5-semialdehyde + NADP + Phosphate, R53: (S)-3-hydroxy-3-methylglutaryl-CoA + 2 NADPH + 2 H ⬄ (R)-mevalonate + CoA + 2 NADP).

During PUFA biosynthesis, NADPH is a necessary cofactor for *de novo* fatty acid synthesis involving desaturation and elongation. In model *i*CY1106, there were 172 reactions involving NADPH (Additional file 6). With ARA production fixed at 0.059 mmol gDW^−1^ h^−1^ (μ = 0.06 h^−1^) and 0.128 mmol gDW^−1^ h^−1^ (μ = 0.03 h^−1^), the flux of NADPH reactions were investigated. In total, 53 NADPH reactions exhibited flux changes of > 10^−6^ mmol gDW^−1^ h^−1^ (Figure 5).

Further research showed that only five reactions (R2, R3, R4, R5, and R6) involving accumulation of NADPH were significantly altered. When ARA production was increased, three reactions (R4, R5, and R6) underwent flux increases, which corresponded to enhanced enzyme activities due to up-regulation of the genes encoding the enzymes. Of these, malic enzyme (ME, EC 1.1.1.40), which catalyzes the conversion of malate to pyruvate (R4), is considered a key enzyme in lipogenesis in *M. alpina*. ME expression and enzyme activity are enhanced during ARA production [43], and the minimization of metabolic adjustment (MOMA) method was used to investigate the role of this enzyme. With the biomass and ARA flux maximized by ME deletion using MOMA, 410 (22.11%) reactions showed flux changes (Additional file 6) compared with the wild type model. The ARA exchange reaction flux was lowered from 0.128 mmol gDW^−1^ h^−1^ to 0.079 mmol gDW^−1^ h^−1^, a decrease of 38.28%. Additionally, all reactions involving NADPH consumption reactions other than R13, R16, R51, and R53 were associated with lipid metabolism. These results indicated that the increase in ARA production was directly correlated with the NADPH consumption rate.

## Conclusions

In this study, a genome-scale metabolic model (*i*CY1106) for *Mortierella alpina* ATCC 32222 was successfully reconstructed using knowledge from the scientific literature and publicly accessible databases. The model comprised 1106 genes, 1854 reactions and 1732 metabolites, and included 247 transport reactions and 216 exchange reactions. Following a series of simulations and verification by measurement of growth rates and substrate usage, the model was found to agree with published literature. The model was used to investigate the effects of the important precursors acetyl-CoA and NADPH on the biosynthesis of PUFAs such as ARA and EPA. FBA results showed that enhancing the pyruvate dehydrogenase complex increased acetyl-CoA availability for increasing ARA production. Limiting the nitrogen source was an effective method for reducing acetyl-CoA consumption, and malic enzyme was found to be a key node in the regulation of NADPH in ARA biosynthesis. Model *i*CY1106 could serve as a useful predictive tool for future systems biology studies to guide the genetic engineering of *M. alpina* to improve the production of industrially important metabolites.

## Methods

### Construction of the *M. alpina* GSMM

The draft model was constructed by amassing reactions from the genome-scale metabolic models of genetically related organisms *Aspergillus terreus* [44], *Pichia pastoris* [45] and *Yarrowia lipolytica* [20]. Reactions were chosen based on orthologs shared between *M. alpina* and the three reference organisms identified by protein sequence similarity searches using BLAST. Protein sequences from *A. terreus* NIH 2624, *P. pastoris* GS 115 and *Y. lipolytica* CLIB 122 were downloaded from UniProt [46]. Open reading frame (ORF) information for *M. alpina* ATCC 32222 was provided by Yong Q. Chen based on sequencing results. The *i*JL1454 GSMM was used as a reference. Additionally, *i*LC915 [45] and *i*YL619_PCP [20] were also used for comparison since both models were concerned with lipid metabolism and overproduction. To ensure accuracy, only sufficiently similar orthologs with e-values ≤10^−30^ and sequence identities ≥40% were included [47]. To expand and update the draft model, the genome of *M. alpina* was re-annotated by submitting ORFs to the KAAS online annotation server [48]. Metabolic reactions absent from the draft model were added from the KEGG database [49] based on KAAS annotation results. MetaCyc [50] and BioPath [51] databases were used to judge reaction reversibility. Compartmentalization information assigned to reactions was determined by subcellular localization prediction tools CELLO [52] and WoLF PSORT. [53] BaCelLo [54] was also used for proteins that were difficult to determine with the other tools. Transport information was obtained by cross-referencing BLATSp searches and the Transporter Classification Database TCDB [55].

To refine the draft model, the gapFind [56] program in the COBRA Toolbox [22] was used to identify metabolic gaps in draft model and literature data were used to fill these gaps. The metabolites in each reaction were characterized based on their chemical formulae and neutral charges, which were obtained using CHEBI [57] and PubChem [58].

### Biomass composition

The biomass equation of *M. alpina* was assumed to have six components: proteins, DNA, RNA, lipids, the cell wall and the small molecule pool [59,60]. Since no detailed information on *M. alpina* DNA and RNA was available, the ratio was assumed to be the same as in the related *Aspergillus niger* [61]. The nucleotide and amino acid composition were calculated based on the *M. alpina* ATCC 32222 genome [2], as no specific experimental data were available. Similarly, the cell wall composition was calculated based on the typical fungal cell wall structure [62]. The lipid composition was calculated based on the current literature [63]. For calculation of energetic parameters, the growth and non-growth associated ATP maintenance values (GAM and NGAM, respectively) were assumed to be the same as those in the central carbon metabolic model of *A. niger* [64]. Detailed biomass composition information can be found in Additional file 7.

### Simulation and analysis

The reconstructed metabolic network was converted into stoichiometric matrix (S = M * N) using the Matlab program, where M represents metabolites and N represents reactions [65]. The basic tools used for model analysis were flux balance analysis (FBA) and flux variability analysis (FVA). GLPK was used for linear programming [22], and Gurobi was used for quadratic programming [66]. *In silico* analysis included growth simulation, gene and reaction essentiality analysis, robustness analysis, and minimization of metabolic adjustment. Analyses were performed according to the instructions for the COBRA Toolbox [22]. Constraints used in each analysis are mentioned in the results section. To analyze model parameters relevant to cell growth, the biomass equation was selected as the objective function. For analysis of ARA production, the exchange reaction of ARA was the objective function.

## Additional files

Additional file 1:
**Detailed information about model** ***i*** **CY1106.**
Additional file 2:
**Shared reactions between Vongsangnak’s model and** ***i*** **CY1106 (not including transport and exchange reactions, no compartments information).**
Additional file 3:
**Result of essential genes identified by FBA using minimum growth medium and yeast extract medium.**
Additional file 4:
**Simulation result for the capability of usage different carbon and nitrogen sources for** ***i*** **CY1106.**
Additional file 5:
**a** **Figure for the robustness analysis about oxygen and ARA.** **b** Figure about the acetyl-CoA consumption pathways in *i*CY1106. **c** Figure for the robustness analysis about glutamate and ARA. Additional file 6:
**FBA simulation result for the reactions involving NADPH at different ARA levels.**
Additional file 7:
**Biomass composition information for** ***M. alpina.***

## Abbreviations

ARA: Arachidonic acid
PUFAs: Polyunsaturated fatty acids
ME: Malic enzyme
GSMM: Genome-scale metabolic model
MNNG: *N*-methyl-*N’*-nitro-*N*-nitrosoguanidine
DGLA: Dihomo-γ-linolenic acid
GLA: γ-linolenic acid
Cb5R: NADH-cytochrome b5 reductase
FBA: Flux balance analysis
EPA: Eicosapentaenoic acid
FVA: Flux variability analysis
MOMA: Minimization of metabolic adjustment
MG: Minimal growth
YE: Yeast extract
DO: Dissolved oxygen
ACC: Acetyl-CoA carboxylase
GAM: Growth associated ATP maintenance
NGAM: Non-growth associated ATP maintenance
