# Supplementary material for: Reconstruction and analysis of a genome-scale metabolic model of the oleaginous fungus Mortierella alpina
Source: BMC Syst Biol. 2015 Jan 13;9:1. doi: 10.1186/s12918-014-0137-8 (PMC4301621; doi:10.1186/s12918-014-0137-8)
Supplement: Additional file 5: — a Figure for the robustness analysis about oxygen and ARA. b Figure about the acetyl-CoA consumption pathways in iCY1106. c Figure for the robustness analysis about glutamate and ARA. [file 12918_2014_137_MOESM5_ESM.docx]

**a: Robustness of ARA production with oxygen absorption rate.** By fixing the glucose uptake rate and cell growth rate, the production of ARA was maximized at each oxygen uptake rates.


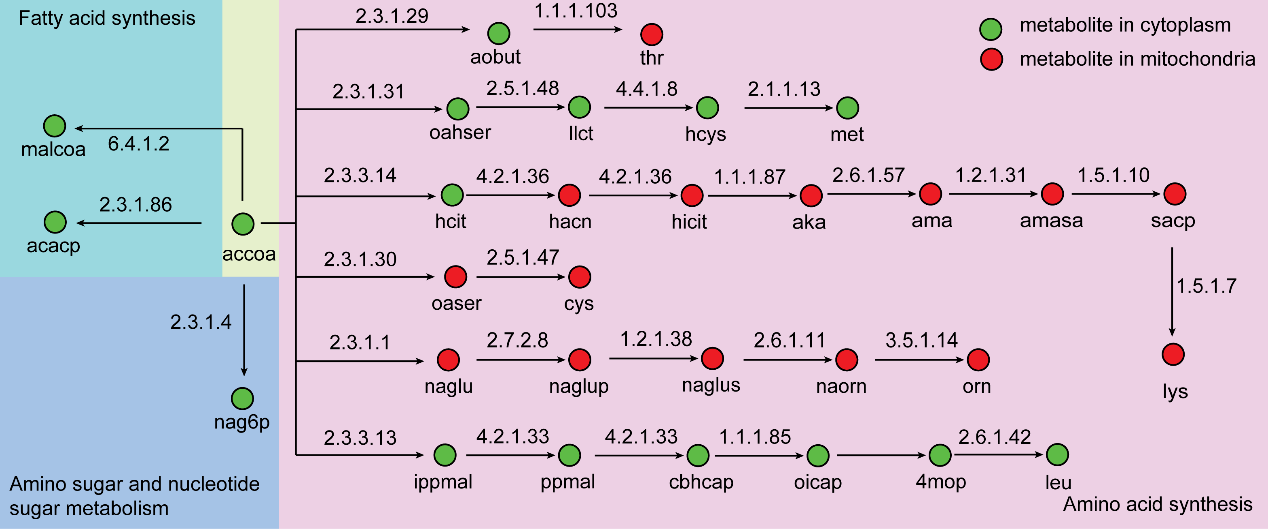


**b: The consumption pathways of acetyl-CoA in *M. alpina***

(acacp: Acetyl-[acyl-carrier protein], malcoa: malonyl-CoA, nag6p: N-Acetyl-D-glucosamine 6-phosphate, aobut: 2-amino-3-oxobutanoate, thr: l-threonine, oahser: o-acetyl-l-homoserine, llct: l-cystathionine, hcys: l-homocysteine, met: l-methionine, hcit: 2-hydroxybutane-1,2,4-tricarboxylate, hacn: but-1-ene-1,2,4-tricarboxylate, hicit: homoisocitratehomoisocitrate，amasa: l-2-aminoadipate 6-semialdehyde, ama: l-2-aminoadipate, aka: 2-oxoadipate, sacp: l-saccharopine, lys: l-lysine, naglu: n-acetyl-l-glutamate, naglup: N-Acetyl-L-glutamyl 5-phosphate, naglus: n-acetyl-l-glutamate 5-semialdehyde, naorn: n2-acetyl-l-ornithine, orn: l-ornithine, ippmal: 2-isopropylmalate, ppmal: 2-isopropylmaleate, cbhcap: (S)-2-Isopropylmalate, oicap: (2s)-2-isopropyl-3-oxosuccinate, 4mop: 4-methyl-2-oxopentanoate, leu: l-leucine)

**c**: **Robustness analysis of ARA production with glutamate uptake rate.** By fixing the glucose uptake rate and cell growth rate, the production of ARA was maximized at each glutamate uptake rates.
